# Vasorelaxant Activity of AP39, a Mitochondria-Targeted H_2_S Donor, on Mouse Mesenteric Artery Rings In Vitro

**DOI:** 10.3390/biom12020280

**Published:** 2022-02-09

**Authors:** Leonardo A. da Costa Marques, Simone A. Teixeira, Flávia N. de Jesus, Mark E. Wood, Roberta Torregrossa, Matthew Whiteman, Soraia K. P. Costa, Marcelo N. Muscará

**Affiliations:** 1 Department of Pharmacology, Institute of Biomedical Sciences, University of Sao Paulo, Sao Paulo 05508-000, SP, Brazil; leomarques996@gmail.com (L.A.d.C.M.); mone@usp.br (S.A.T.); flavia.netodejesus@ucalgary.ca (F.N.d.J.); skcosta@usp.br (S.K.P.C.); 2 Medical School, University of Exeter, Exeter EX1 2LU, UK; M.E.Wood@exeter.ac.uk (M.E.W.); r.torregrossa@ex.ac.uk (R.T.); m.whiteman@exeter.ac.uk (M.W.); 3 School of Biosciences, University of Exeter, Exeter EX4 4QD, UK; 4 Department of Physiology and Pharmacology, Cumming School of Medicine, University of Calgary, 3330 Hospital Dr. NW, Calgary, AB T2N 4N1, Canada

**Keywords:** hydrogen sulfide, mesenteric artery, vasorelaxation, mitochondria

## Abstract

Mitochondria-targeted hydrogen sulfide (H_2_S) donor compounds, such as compound AP39, supply H_2_S into the mitochondrial environment and have shown several beneficial in vitro and in vivo effects in cardiovascular conditions such as diabetes and hypertension. However, the study of their direct vascular effects has not been addressed to date. Thus, the objective of the present study was to analyze the effects and describe the mechanisms of action of AP39 on the in vitro vascular reactivity of mouse mesenteric artery. Protein and gene expressions of the H_2_S-producing enzymes (CBS, CSE, and 3MPST) were respectively analyzed by Western blot and qualitative RT-PCR, as well the in vitro production of H_2_S by mesenteric artery homogenates. Gene expression of CSE and 3MPST in the vessels has been evidenced by RT-PCR experiments, whereas the protein expression of all the three enzymes was demonstrated by Western blotting experiments. Nonselective inhibition of H_2_S-producing enzymes by AOAA abolished H_2_S production, whereas it was partially inhibited by PAG (a CSE selective inhibitor). Vasorelaxation promoted by AP39 and its H_2_S-releasing moiety (ADT-OH) were significantly reduced after endothelium removal, specifically dependent on NO-cGMP signaling and SK_Ca_ channel opening. Endogenous H_2_S seems to participate in the mechanism of action of AP39, and glibenclamide-induced K_ATP_ blockade did not affect the vasorelaxant response. Considering the results of the present study and the previously demonstrated antioxidant and bioenergetic effects of AP39, we conclude that mitochondria-targeted H_2_S donors may offer a new promising perspective in cardiovascular disease therapeutics.

## 1. Introduction

Hydrogen sulfide (H_2_S) is an endogenous gaseous transmitter first found in neural tissues in 1996 by Abe and Kimura [1], and a plethora of effects in mammals have been described since then, including its cardiovascular actions [2,3]. The major sources of endogenous H_2_S production rely on the enzyme activity of cystathionine γ-lyase (CSE), cystathionine β-synthase, and 3-mercaptopyruvate sulfurtransferase (3MPST; [3,4]), although there are also nonenzymatic pathways of H_2_S production in the bloodstream [5]. Reduced H_2_S production in CSE-knockout mice leads to hypertension [6] and atherosclerosis [7], thus seeming evident that the lack of H_2_S production contributes to cardiovascular diseases, particularly impaired vascular tone control. In this way, increasing H_2_S bioavailability may represent a useful therapeutic strategy for these conditions.

One of the first described vascular effects of H_2_S was the vasorelaxation of mouse aorta [2] via potassium channels, either of the type ATP-sensitive (K_ATP_), small conductance calcium-activated (SK_Ca_), and voltage-gated (K_v_) potassium channels, in addition to nitric oxide synthase (NOS) activation [8,9,10,11,12,13,14,15]. Moreover, in resistance vessels, such as those of the mesenteric vascular bed, H_2_S acts as an endothelium-derived hyperpolarization factor (EDHF [16]). These H_2_S effects are mediated by S-sulfhydration of target proteins, a covalent conversion of cysteine free thiol groups with low pKa into persulfide groups; this reaction has been reported to occur in the activation of K^+^ channels, primarily K_ATP_ [17].

CSE and 3MPST are the major sources of H_2_S production in resistance vessels, including the mesenteric bed [18]. In addition to vasorelaxation, CSE-derived H_2_S can play other beneficial roles, such as an antioxidant agent in endothelial cells [19] and via its bioenergetics effects [20], especially under hypoxia [21]. S-sulfhydration of mitochondrial ATP-synthase represents one of the physiological bioenergetic effects promoted by CSE-derived H_2_S, as demonstrated in the liver and kidney-derived cell lines, which were absent in livers from CSE^−/−^ mice [22]. However, the actual relevance of this mitochondrial path in vascular tissues still remains to be fully understood.

It is generally agreed that the beneficial effects of H_2_S are observed within the nanomolar range since toxic effects start to appear at H_2_S concentrations within the micromolar range due to inhibition of mitochondrial complex IV [23]. As shown by Lagoutte and colleagues (2010), H_2_S has a substantial role in mammalian smooth muscle cell bioenergetics, acting as a sulfide quinone reductase (SQR) substrate, thus stimulating mitochondrial chain electron transport [19].

In fact, mitochondria-targeted H_2_S donors have been shown to exert beneficial effects on bioenergetic parameters in vitro via enhancement of maximal respiration rate and sparing respiration capacity, in addition to their antioxidant effects [24,25]. For example, studies with the mitochondria-targeted H_2_S donor, AP39 [(10-oxo-10-(4-(3-thioxo-3H-1,2-dithiol-5yl) phenoxy) decyl) triphenyl phosphonium bromide], have shown concentration-dependent effects on mitochondrial metabolism of endothelial cells, including protective effects against glucose-induced oxidative stress (such as reduced damage to mitochondrial DNA [24,25]), as well as in epithelial cells [26]. Furthermore, endothelial cell senescence was partially reversed by AP39 via interference on alternative splicing [27]. Other in vitro antioxidant effects promoted by AP39 have also been described in cultured cardiomyocytes exposed to H_2_O_2_ [28].

In vivo results show that treatment with AP39 resulted in reduced heart infarct size in rats submitted to ischemia–reperfusion [29], as well as vasodilation in anesthetized rats [30].

Considering the lack of studies on the direct vascular effects of mitochondria-targeted H_2_S donors, in the present study, we aimed to investigate the in vitro vascular effects of AP39 on mouse mesenteric resistance artery rings and the underlying mechanisms.

## 2. Materials and Methods

### 2.1. Animals

All the experimental procedures were approved by the local ethics committee (CEUA/ICB 7759060218, approval date: 6 February 2018), in accordance with both the CONCEA (National Council of Control in Animal Experimentation) and the ARRIVE guidelines. Male SPF C57BL/6 mice (23 ± 1 g, 8–10 weeks old) were supplied by the Facility for SPF mice production at the USP Medical School Animal Facility Network (University of São Paulo). The animals were housed under controlled environmental conditions (12:12-h light-dark cycle; 22 ± 2 °C) with free access to standard rodent chow and filtered tap water.

### 2.2. In Vitro Vascular Response

After anesthesia with ketamine (80 mg/kg) and xylazine (20 mg/kg), the animals were exsanguinated, the mesenteric bed was quickly harvested and placed in ice-cold Krebs-Henseleit solution (in mM: 130 NaCl, 4.7 KCl, 14.9 NaHCO_3_, 1.6 CaCl_2_·2H_2_O, 1.18 KH_2_PO_4_, 1.17 MgSO_4_·7 H_2_O, 0.026 EDTA, and 5.5 glucose). After removing perivascular and adjacent connective tissue, first-order mesenteric arteries were cut in 2 mm-length rings and placed in the wire myograph chambers (Danish Myo Technology - DMT, Hinnerup, Denmark) containing Krebs-Henseleit solution (pH 7.4) continuously bubbled with 95/5 O_2_/CO_2_ (*v*/*v*) at 37 °C.

Two tungsten wires (40 µm diameter) were passed through the ring’s lumen, one of them being attached to a force-measurement transducer and the other driven by a micrometer. After an equilibration period (30 min), the wall tension was set to a value corresponding to an intravascular pressure of 100 mmHg (according to the “Normalization Module” specifications; DMT, Hinnerup, Denmark). After a new equilibration period at the set resting tension, the rings were contracted with 120 mM KCl in order to assess their smooth muscle viability. The viability of the vascular endothelium was assessed by its response to 10 µM acetylcholine after contraction with 1 µM phenylephrine (those rings relaxing less than 80% of the phenylephrine-induced contractile tonus were discarded). In some rings, the endothelium was mechanically removed by friction of the mounting wires over the inner arterial wall, and this procedure was considered appropriate if the relaxing response to acetylcholine was less than 20% of the phenylephrine-induced contractile tonus.

To assess the vasoactive effects of AP39 and 5-(4-hydroxyphenyl)-3H-1,2-dithiole-3-thione (ADT-OH, the H_2_S donor core of AP39) on resting tension, the rings were exposed to increasing (cumulative) concentrations of the compounds (in the range 0.01 to 30 nM).

In order to assess the vasorelaxation of the H_2_S donors, the rings were precontracted with phenylephrine (at concentrations within the range 1–2 µM), in order to achieve 70% of the maximal response produced by 120 mM KCl, and the concentration–response curves were performed with AP39 and ADT-OH as described above. In some experiments, the rings were preincubated (for 30 min) with different inhibitors or blockers: 10 μM indomethacin (a nonselective COX cyclooxygenase inhibitor), 100 μM L-N^G^-nitroarginine methyl ester (L-NAME; a nonselective nitric oxide synthase inhibitor), 10 μM 1H-[1,2,4]oxadiazolo[4,3-a]quinoxalin-1-one (ODQ; a soluble guanylate cyclase inhibitor), 10 nM sildenafil (a type V-phosphodiesterase inhibitor), 10 mM aminooxyacetic acid (AOAA; a nonselective H_2_S producing enzyme inhibitor), 3 mM tetraethylammonium (TEA; a nonselective K^+^ channel blocker), 10 μM glibenclamide (an ATP-sensitive K^+^ channel blocker) or 5 μM apamin (a Ca^2+^-activated K^+^ channel blocker). Relaxation was expressed as a percentage of the reduction in phenylephrine-induced contraction, and the parameters E_max_ (maximal effect) and pA_2_ (potency; necessary concentration to achieve 50% of maximal effect) were obtained from each individual concentration vs. response curve plotted by nonlinear regression fit.

### 2.3. Materials

AP39 was synthesized in-house, as previously described [31]. All other drugs and reagents were obtained from Sigma-Aldrich (St. Louis, MO, USA), except for apamin (Bio-Techne, Abingdon, UK). Stock solutions of the H_2_S donors AP39 and ADT-OH were prepared at 10 mM in 100% dimethylsulfoxide (DMSO; Labsynth, Diadema, Brazil), kept at −80 °C, and diluted to the final concentrations with Krebs-Henseleit solution just before use. All other compound solutions were freshly prepared just before use at concentrations 1000-fold higher than the final used concentrations. Apamin, glibenclamide, ODQ, and sildenafil were firstly dissolved in 100% DMSO; L-NAME, TEA, and AOAA were dissolved in distilled water, and indomethacin was dissolved in 10% Na_2_CO_3_.

### 2.4. H_2_S Producing Enzyme Expression

Protein expression of the H_2_S producing enzymes (CSE, CBS, and MPST) in mouse mesenteric artery homogenates was analyzed by Western blotting as previously described [32]. Briefly, 10 µg of total proteins from homogenates were separated by 10% SDS-PAGE electrophoresis. The electro-transferred nitrocellulose membranes from gels were incubated in primary antibodies directed against CSE (1:1500 polyclonal rabbit IgG1 anti-mouse CSE; Proteintech, Rosemont, IL, USA), CBS (1:1500 monoclonal mouse IgG anti-mouse CBS; Abnova, Taipei, Taiwan), or 3MPST (1:1000 polyclonal mouse IgG anti-mouse MPST; Abnova). After proper washing, the membranes were incubated with the corresponding secondary antibodies (1:3000 polyclonal anti-rabbit IgG or monoclonal anti-mouse IgG coupled to horseradish peroxidase; Bio-Rad, Hercules, CA, USA). Immunoreactive bands were detected by chemiluminescence (resultant from the reaction of HRP with the ECL substrate solution kit), using the ChemiDocTM MP image acquisition system (Bio-Rad, Hercules, CA, USA).

Gene expression of the H_2_S producing enzymes was analyzed by qualitative polymerase chain reaction after reverse transcription (RT-PCR), as previously described [33]. Briefly, total RNA from mouse mesenteric arteries, brain, and liver were extracted using the TRIzol reagent according to the manufacturer’s instructions (Life Technologies, Carlsbad, CA, USA). cDNA was synthesized from 180 ng of total RNA using RT enzyme (200 U, M-MLV reverse transcriptase; Promega, Madison, WI, USA) according to the manufacturer’s protocol. PCR reactions were performed for specific primer amplification of CSE (forward: GCA CAA ATT GTC CAC AAA CG; reverse: GTC CTT CTC AGG CAC AGA GG; amplicon size: 573 bp), CBS (forward: CTT GGA CAT GCA CTC AGA AAA G; reverse: TGA TAG TGT CTC CAG GCT TCA A; amplicon size: 365 bp), 3MPST (forward: ATG CCC CAA GAG GAG AAA GT; reverse: TAG GCA GCA TGT GGT CGT AG; amplicon size: 381 bp) and for the internal control glyceraldehyde-3-phosphate dehydrogenase-GAPDH (forward: GGT GCT GAG TAT GTC GTG GA; reverse: TTC AGC TCT GGG ATG ACC TT; amplicon size: 400 bp). PCR reaction products (*n* = 4) were electrophoresed on 3% ethidium bromide-stained 1.5% agarose gels. Gel images were captured under UV light using the ChemiDoc™ MP Imaging System (Bio-Rad, Hercules, CA, USA).

### 2.5. In Vitro H_2_S Production

The in vitro H_2_S production by homogenates of mouse mesenteric artery, heart, and thoracic aorta was analyzed by the method of lead sulfide formation [34] with some modifications. Briefly, the tissues were excised, homogenized (in phosphate buffer 100 mM, pH 7.4, containing 1 mM PMSF, 10 µg/mL leupeptin, 10 µg/mL trypsin inhibitor and 2 µg/mL aprotinin), and centrifuged (10,000× *g*, 10 min). Using a 96-well microplate, the supernatants (equivalent to 0.2 mg/mL protein diluted with 100 mM phosphate buffer, pH 7.4) were mixed with the substrate (10 mM L-cysteine) and the enzyme cofactor (2 mM 5′-phosphate pyridoxal-5′-phosphate). The plate was covered with a filter paper that had previously been embedded with 100 mM lead acetate and allowed to dry and incubated for 3 h at 37 °C. At the end of the incubation period, the optical densities of the dark spots formed on the filter paper (due to the formation of a dark brown PbS precipitate) were analyzed and quantified using the software ImageLabTM (Bio-Rad, Hercules, CA, USA). The production of hydrogen sulfide from each sample was calculated by extrapolation from a NaHS standard curve (15.6–500 μM). In order to pharmacologically characterize the enzymatic source of H_2_S, this generation was also analyzed after the incubation of the tissue supernatants with the H_2_S producing enzyme inhibitors DL-propargylglycine (PAG, a preferential CSE inhibitor) or aminooxyacetic acid (AOAA, a nonselective H_2_S producing enzyme inhibitor), both at 10 mM, for 30 min at 37 °C.

### 2.6. Statistical Analysis

Data and statistical analysis comply with the recommendations of experimental design and analysis in pharmacology [35]. Data are expressed as mean ± S.E.M; n indicates the number of independent animals per group. Differences among the different group means were analyzed by either the Student’s *t*-test for unpaired observations or by one-way ANOVA followed by the Bonferroni post hoc test for multiple comparisons, as appropriate, using the software GraphPad Prism (version 6.01; GraphPad Software, Inc., San Diego, CA, USA). Differences between group means with the value of *p* < 0.05 were considered statistically significant.

### 2.7. Data Availability

Data sets originally produced by the current study are available from the corresponding author upon reasonable request.

## 3. Results

### 3.1. Expression of H_2_S-Producing Enzymes

**Figure 1:**
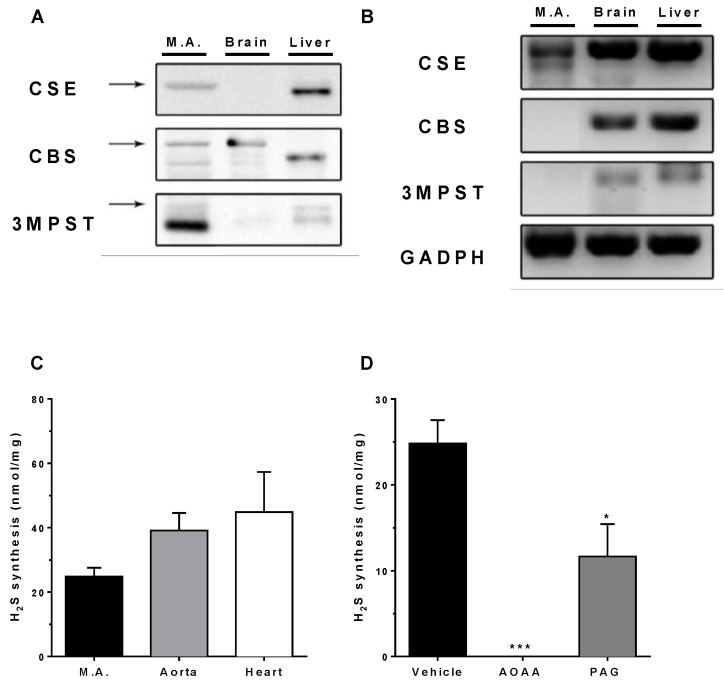
Characterization of the expression and activity of endogenous H_2_S-producing enzymes CSE, CBS, and 3MPST in mouse mesenteric artery. Panels (**A**,**B**): protein and gene expression, respectively (*n* = 4/group). Panel (**C**): In vitro H_2_S production by homogenates of mouse mesenteric arteries, aorta, and heart (*n* = 5/group). Panel (**D**): Inhibition of in vitro H_2_S generation by mesenteric artery homogenates by 10 mM PAG or AOAA (*n* = 5/group). Data are represented as mean ± S.E.M. * *p* < 0.05 and *** *p* < 0.001 vs. Vehicle, as analyzed by one-way ANOVA followed by the Bonferroni post hoc test for multiple comparisons.

As shown in Figure 1 (panel A), Western blot analysis revealed the protein expression of the three studied H_2_S-producing enzymes in the mesenteric artery homogenates (*n* = 4; liver and brain homogenates were used as positive controls for CSE and CBS, respectively). On the other hand, RT-PCR analysis showed the presence of CSE mRNA in the vessels, whereas only little 3MPST or CBS mRNA expressions were found (Figure 1, panel B). Complete Western Blot membranes and RT-PCR gels are included in the Supplementary Material (Figure S1).

### 3.2. In Vitro H_2_S Generation by Mesenteric Artery Homogenates

In addition to mouse mesenteric artery homogenates, thoracic aorta and heart homogenates were also analyzed for their endogenous H_2_S production in vitro. No statistically significant differences were observed among the studied tissues (*n* = 5; Figure 1, panel C). As shown in Figure 1 panel D, H_2_S production by mesenteric artery homogenates was completely abolished by 10 mM AOAA (*p* < 0.01), while 10 mM PAG caused a partial (50%) inhibition (*p* < 0.05).

### 3.3. In Vitro Vascular Effects of AP39 and ADT-OH

**Figure 2:**
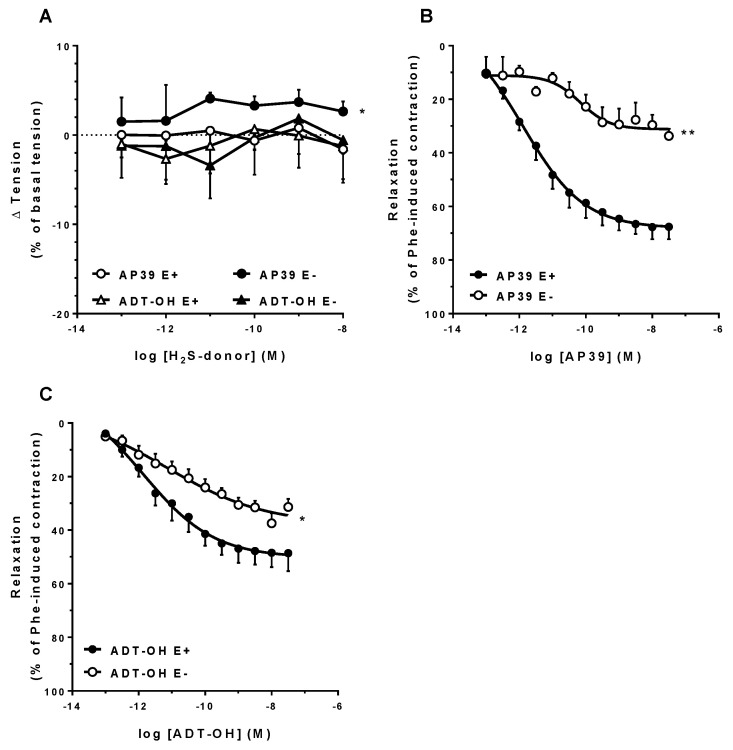
In vitro effects of the H_2_S donors AP39 and ADT-OH on mouse mesenteric artery rings. Panel (**A**) shows the effects of AP39 or ADT-OH on resting tension, as analyzed in both intact and mechanically endothelium-denuded rings. Panels (**B**,**C**) show the vasorelaxant effects of AP39 and ADT-OH, respectively, on Phe precontracted rings, either intact (E+) or after the mechanical removal of the endothelial layer (E−). * *p* < 0.05 and ** *p* < 0.01 vs. E+ E_max_ (as analyzed by the Student’s *t*-test for unpaired data; *n* = 5–7/group).

Figure 2 (panel A) shows the concentration-related effects of AP39 on the resting tension of the mesenteric artery rings. Over the range 0.1 pM–10 nM, neither AP39 nor ADT-OH exerted any significant effect on vascular tone, although AP39 induced a significant contraction when the endothelium was mechanically removed (E− E_max_: 4.6 ± 1.2% vs. E+ E_max_: 1.0 ± 0.7%, *p* < 0.05, *n* = 5).

After precontraction of the rings with Phe, AP39 caused a concentration-related relaxation of the intact rings (E+ E_max_: 72.5 ± 4.6%, pA_2_: 12.2 ± 0.4, *n* = 7; Figure 2, panel B), which was significantly attenuated in the endothelium-denuded rings (E− E_max_: 34.6 ± 3.1%, *p* < 0.01; pA_2_: 10.0 ± 0.6, *p* < 0.05; *n* = 5). ADT-OH-induced relaxation followed a similar endothelium-dependent behavior (E+ E_max_: 50.4 ± 5.8% vs. E−: 36.8 ± 2.2%, *n* = 7, *p* < 0.05, Figure 2C), although of lower efficacy in comparison with AP39 (*p* < 0.05). Representative recordings of AP39 and ADT-OH relaxing responses are included in the Supplementary Material (Figure S2).

**Figure 3:**
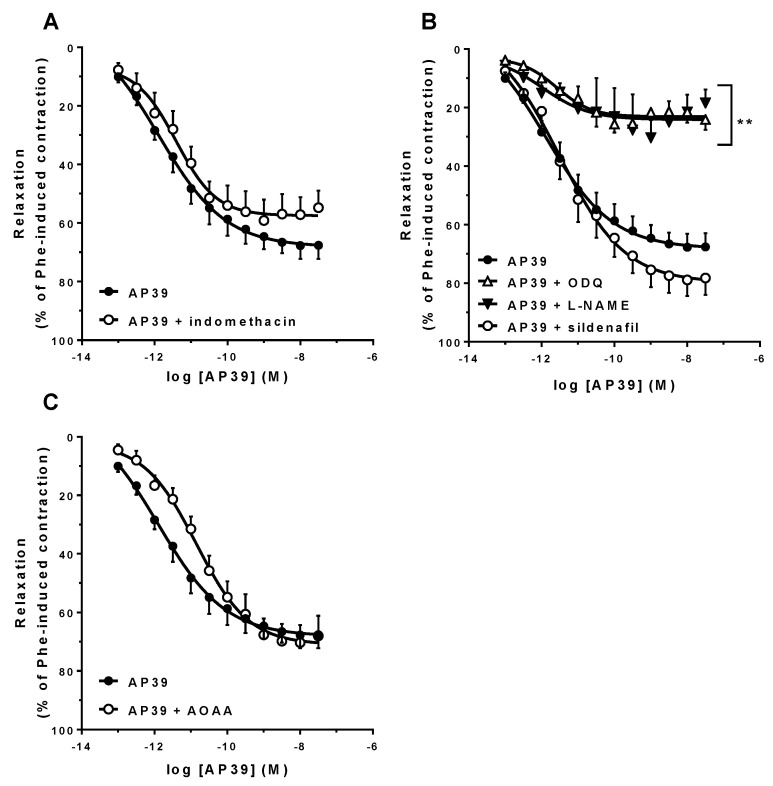
Involvement of endogenous COX, NO, and H_2_S pathways in the AP39-induced vasorelaxation of mouse mesenteric artery rings precontracted with Phe. The responses were evaluated in the presence of either 10 µM indomethacin (panel (**A**); *n* = 5/group), inhibitors of the NO-cGMP signaling pathway (100 µM L-NAME, 10 µM ODQ or 5 µM sildenafil; panel (**B**); *n* = 5–7/group) or 10 mM AOAA (panel (**C**); *n* = 7/group). Data are expressed as mean ± S.E.M. Emax differences were observed (** *p* < 0.01 vs. E+), as analyzed by the Student’s *t*-test.

As shown in Figure 3, the presence of either 10 µM indomethacin (panel A) or 5 nM sildenafil (panel B) in the tissue bath did not affect the vasorelaxant response of the Phe-precontracted rings to AP39. However, the presence of 100 µM L-NAME or 10 µM ODQ significantly attenuated AP39 induced vasorelaxation (AP39 + L-NAME E_max_: 23.9 ± 5.1%, *n* = 5, *p* < 0.001; AP39 + ODQ E_max_: 22.9 ± 3.4%, *n* = 6, *p* < 0.001; Figure 3, panel B). Inhibition of the H_2_S-producing enzymes by 10 mM AOAA resulted in significant loss of AP39 vasorelaxant potency (AP39 + AOAA pA_2_ = 11.0 ± 0.3, *n* = 7, *p* < 0.05, Figure 3C) with no significant effects on E_max_.

**Figure 4:**
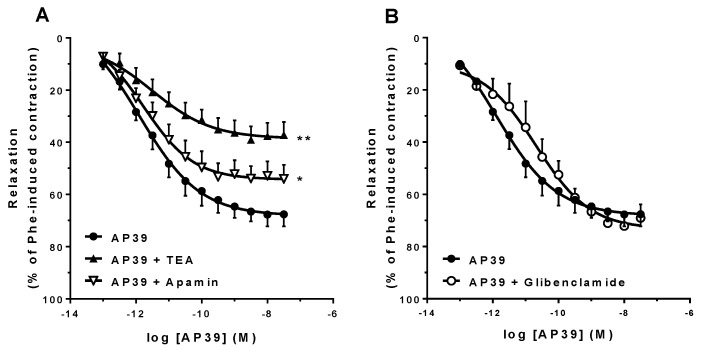
Participation of K^+^ channels in the AP39-induced vasorelaxation of mouse mesenteric artery rings precontracted with Phe. The responses were evaluated in the presence of the nonselective K^+^ channel blocker TEA (3 mM) or the selective SK_Ca_ channel blocker apamin (5 µM; panel (**A**); *n* = 7/group). Panel (**B**) shows the AP39 responses in the presence of the K_ATP_ channel blocker glibenclamide (10 µM; *n* = 5/group). Data are expressed as mean ± S.E.M. E_max_ differences were observed (* *p* < 0.05 or ** *p* < 0.01 vs. AP39 alone) as analyzed by the Student’s *t*-test.

Figure 4 (panel A) shows that in the presence of 3 mM TEA or 5 µM apamin, the vasorelaxant activity of AP39 was significantly attenuated (AP39 + TEA E_max_: 38.5 ± 5.2%, *n* = 7, *p* < 0.01; AP39 + apamin E_max_: 52.6 ± 5.9%, *n* = 5, *p* < 0.05). On the other hand, 10 µM glibenclamide did not affect AP39-induced vasorelaxation (Figure 4, panel B).

For the sake of clarity, all the E_max_ and pA_2_ results are summarized in Table 1.

## 4. Discussion

Vascular relaxation elicited by H_2_S donors has already been described in several in vitro studies [6,8,9,11,16,36]. However, most of them employed inorganic sulfide salts (e.g., NaHS or Na_2_S) as a source of H_2_S, in which case, H_2_S is one of the species in equilibrium as a direct function of pH. Therefore, the free H_2_S concentrations spontaneously achieved with these salts are often well above the physiological H_2_S concentrations. On the other hand, the mitochondria-targeted H_2_S donor AP39 can continuously release H_2_S at a controlled rate [25]. Tomasova et al. have previously assessed the in vivo hemodynamic effects of AP39 in hypertensive rats, which included a transient reduction in blood pressure and heart rate, these effects being mediated by cardiac membrane Ca^2+^ and Cl^−^ channels [30]. However, no studies have addressed to date the direct effects of this compound on vascular reactivity, particularly on resistance vessels (such as the mesenteric artery).

Resistance arteries are the main vessels responsible for the control of blood flow, and consequently blood pressure, due to their small internal diameter and the thick muscular wall in relation to the narrow lumen [8,37]. In this way, investigations on the vascular effects of new compounds on resistance vessels are a mandatory step for the development of novel cardiovascular drugs.

In the present study, we showed that resistance mesenteric arteries express all three H_2_S-producing enzymes, CSE being the most abundant, with a minor expression of both CBS and 3MPST, in agreement with previous reports [38] and confirming the relevant physiological role of CSE in the control of mesenteric blood flow [37,39]. Although CSE and 3MPST play major roles in the cardiovascular system (they are also the main enzymes expressed in vessels such as the coronary artery [40] and aorta [41]), some studies performed on human endothelial cells from an umbilical vein (HUVEC) point out that CBS activity can be involved in endothelial function regulation [42,43]. H_2_S production has thus several roles along the cardiovascular system, such as control of vascular tonus [6,39,44] and angiogenesis [45]. We have observed that H_2_S-production by mesenteric artery homogenates in vitro was partially inhibited by the CSE inhibitor PAG and completely abolished by AOAA, a compound formerly considered as a selective CBS inhibitor, although it was later shown to be a nonselective inhibitor of the H_2_S-producing enzymes [46].

Both AP39 and its H_2_S-releasing moiety (ADT-OH) failed to significantly alter the basal tension of intact mesenteric arteries rings; however, AP39, but not ADT-OH, caused a slight (although significant) tension increase when the endothelium layer was mechanically removed (Figure 2A). To our knowledge, there are no studies to date showing the direct effects of AP39 on vascular smooth muscle (VSM). Despite the well-documented relaxing effects of VSM by H_2_S, the observed vasoconstrictor effects of AP39 should not be related to H_2_S release but rather to the presence of the mitochondrial addresser triphenylphosphonium-TPP^+^ moiety in the AP39 structure. Indeed, Trnka et al. (2015) have reported the negative impact of hydrophobic TPP^+^ derivatives on mitochondrial membrane potential and respiratory chain activity [47]; however, additional experiments are needed in order to validate this hypothesis in the mouse mesenteric artery smooth muscle.

On the other hand, when the mesenteric artery rings were precontracted with Phe, AP39 potently relaxed the vessels in an endothelium-dependent manner. ADT-OH also caused vasorelaxation, albeit to a lesser extent, in terms of potency, efficacy, and endothelium dependency (Figure 2, panels B and C). The vasorelaxant properties of AP39 were also observed in rat mesenteric artery rings (prepared from third-order branches; included in the Supplementary Material; Figure S3).

The high sensitivity of the studied vessel to AP39 is noteworthy (pA_2_ = 12.2 ± 0.4). Although H_2_S concentrations along the cardiovascular system depend on cell types, pathological condition and species [48,49], the vasorelaxant effects of AP39 were observed at concentrations that resemble those of free H_2_S under physiological conditions (i.e., within the low nM range; [3]), and are in agreement with the AP39 concentrations that result in protective effects on cultured endothelial cells submitted to oxidative stress [25], as AP39 concentrations above 300 nM lead to significant cell death.

The relevance of endothelium in H_2_S-elicited vascular relaxation is well established [2], and thus, the residual response of AP39 and ADT-OH in the absence of endothelium should be related to direct interactions with VSM cells via Ca^2+^ channel closure, as demonstrated on cardiac membranes [30]. In this paper, the authors also show the involvement of NO-signaling in the AP39 actions in vivo, and our results confirm this involvement in the AP39-induced vasorelaxation of mesenteric resistance artery rings in vitro, as neither NOS inhibition by L-NAME, nor sGC inhibition by ODQ significantly reduced AP39 effects to the same extent (Figure 3B), and similar to that observed after endothelium removal (Figure 2B).

As is also shown in Figure 3B (and Table 1), the presence of sildenafil did not result in significant additive or synergic effects with those of AP39 alone, thus suggesting that AP39 could also be inhibiting type-V phosphodiesterase (PDE), as was already shown in rat aorta for endogenous H_2_S or exogenous NaHS at nanomolar concentrations [44].

As shown in Figure 3C and Table 1, when endogenous H_2_S production was inhibited by AOAA, a significant loss of AP39 vasorelaxant potency was observed. As previously shown by Coletta et al. (2012) in mouse aorta rings [13], this effect is related to the lower production of endothelial NO secondary to inhibition of H_2_S production via interference with Ca^2+^-dependent pathways in endothelial cells [16,50,51].

Due to its chemical nature, AP39 can accumulate in the mitochondrial environment (up to 500-fold; [23]). In this way, under AOAA inhibition of the cytosolic H_2_S sources that maintain endothelial NO production, the low cytosolic concentrations of AP39-derived H_2_S cannot compensate for this NO reduction. This hypothesis is further supported by our results that show that AOAA does not interfere with the vasorelaxant response of the mesenteric artery rings to the NO donor sodium nitroprusside, while the endothelial-dependent relaxation response of these vessels to acetylcholine is attenuated (included in the Supplementary Material; see Figure S4).

As opposed to AP39, the H_2_S-releasing compound ADT-OH is devoid of mitochondrial effects in the nM concentration range [24]. In addition, and as shown in Figure 2, the vasorelaxation elicited by ADT-OH was of lower intensity in comparison with the response to AP39. As a whole, we can thus conclude that mitochondrial-dependent components are involved in the vasorelaxant response to AP39. Testai and coworkers (2016) demonstrated that in isolated rat cardiac mitochondria, H_2_S could partially depolarize the mitochondrial membrane potential via K_ATP_ channels [52]. In turn, this depolarization can lead to eNOS activation (via PI3K-Akt) and NO-dependent vasorelaxation, as shown in isolated cerebral arteries [53].

In Figure 4A, it is shown that AP39 vasorelaxation is attenuated in the presence of the nonspecific K^+^ channel blocker TEA, although selective K_ATP_ inhibition by glibenclamide did not affect the AP39 response (Figure 4B). Activation of K_ATP_ in VSM membrane cells was one of the first vascular targets described for H_2_S effects [18]; however, this membrane channel does not seem to be involved in the AP39-induced mesenteric artery relaxation. On the other hand, in relation to mitochondrial K_ATP_ channels, as mentioned above, they could be targeted by AP39-derived H_2_S and, in turn, cause relaxation via eNOS-derived NO production. Although glibenclamide can inhibit both membrane and mitochondrial K_ATP_ channels [54], it is important to emphasize that its mitochondrial effects were always studied in isolated mitochondria, thus raising doubts regarding its mitochondrial availability when the whole intact cell is exposed to the compound.

Regarding the Ca^2+^-dependent K^+^ channels SK_Ca_, several previous studies have associated these channels with exogenous H_2_S-induced vasorelaxation [8,9,10], and this seems to be also the case for AP39-induced mesenteric artery relaxation, as, in the presence of apamin, this response was significantly attenuated (Figure 4A). Furthermore, in addition to the canonical NO-cGMP pathway, endothelial NO-induced vasorelaxation can also involve VSM cell hyperpolarization via membrane SK_Ca_ channel activation [55], and hence, it is possible that AP39 may activate these channels both directly and indirectly (via induction of endothelial NO synthesis). These hypotheses are under current investigation.

Oxidative stress plays a central role as an etiological factor of endothelial dysfunction in most cardiovascular diseases by interfering with many intracellular pathways [56], including H_2_S signaling [6]. Gerő et al. (2016) showed that low nanomolar AP39 concentrations were able to control hyperglycemia-induced oxidative stress damage in cultured endothelial cells, and the mechanisms involved antioxidant activity and reversal of the cellular bioenergetic failure due to mitochondrial membrane hyperpolarization [25]. These beneficial antioxidant effects and cellular bioenergetic improvements by AP39 were confirmed by others, not only in cultured endothelial cells but also in cardiomyocytes exposed to oxidative stress [24,27,28]. It is thus evident that the conjunction of the antioxidant and vasorelaxant properties of the mitochondria-targeted H_2_S donor AP39 represents an attractive model for a new compound class aimed at the pharmacological treatment of cardiovascular diseases [57,58,59].

## 5. Conclusions

Previous studies have demonstrated the in vitro and in vivo beneficial effects of AP39; however, the direct vascular effects of this compound are to date unknown. AP39-induced vasorelaxation depends on NO signaling and K^+^ channels activation. The AP39 mechanism of action is partially similar to other H_2_S donors’ signaling; however, the activation of VSM membrane K_ATP_, the first described mechanism of action of H_2_S in the cardiovascular system, is not involved, although the involvement of the mitochondrial K_ATP_ channel cannot be excluded. In addition to the well-documented antioxidant and bioenergetic effects of AP39, the results shown herein stimulate future investigations aiming at its application as a novel therapeutic agent for cardiovascular diseases.

## Figures and Tables

**Table 1:** Summary of the AP39 concentration–response curve parameters obtained from the AP39-induced vasorelaxation of mouse mesenteric artery rings under control conditions and in the presence of the different enzyme inhibitors and channel blockers tested (shown in Figure 2, Figure 3 and Figure 4). * *p* < 0.05; ** *p* < 0.01 vs. intact rings with no additions (E+).

Protocol	E_max_ (%)	pA_2_	*n*
E+	72.5 ± 4.6	12.2 ± 0.4	7
E−	34.6 ± 3.1 **	10.0 ± 0.6 **	5
10 µM indomethacin	57.1 ± 6.3	11.6 ± 0.4	7
100 µM L-NAME	23.9 ± 5.1 **	10.5 ± 0.7 *	5
10 µM ODQ	22.9 ± 3.3 **	11.3 ± 0.2	7
5 nM sildenafil	81.0 ± 5.7	11.7 ± 0.3	7
10 mM AOAA	72.8 ± 6.4	11.0 ± 0.3 *	7
3 mM TEA	38.6 ± 5.2 **	11.5 ± 0.3	7
10 µM glibenclamide	72.6 ± 4.2	11.2 ± 0.6	5
5 µM apamin	52.0 ± 4.9 *	11.5 ± 0.4	7

## Data Availability

The data presented in this study are available on request from the corresponding author.

## Supplementary Materials

The following supporting information can be downloaded at: https://www.mdpi.com/article/10.3390/biom12020280/s1, Figure S1: Complete Western blot membranes and RT-PCR gels, Figure S2: Representative electronic recordings of the vasorelaxant responses induced by AP39 and ADT-OH on mouse mesenteric artery rings precontracted with Phe, Figure S3: Rat mesenteric artery ring (3rd-order branch) response to AP39 and ADT-OH after after Phe-induced constriction, Figure S4: Mouse mesenteric artery ring responses to different vasoactive compounds.
