# Supplementary material for: Vasorelaxant Activity of AP39, a Mitochondria-Targeted H2S Donor, on Mouse Mesenteric Artery Rings In Vitro
Source: Biomolecules. 2022 Feb 9;12(2):280. doi: 10.3390/biom12020280 (PMC8961640; doi:10.3390/biom12020280)
Supplement: Supplementary file 1 [file biomolecules-12-00280-s001.zip › biomolecules-1547512-supplementary.pdf]

Supplementary material

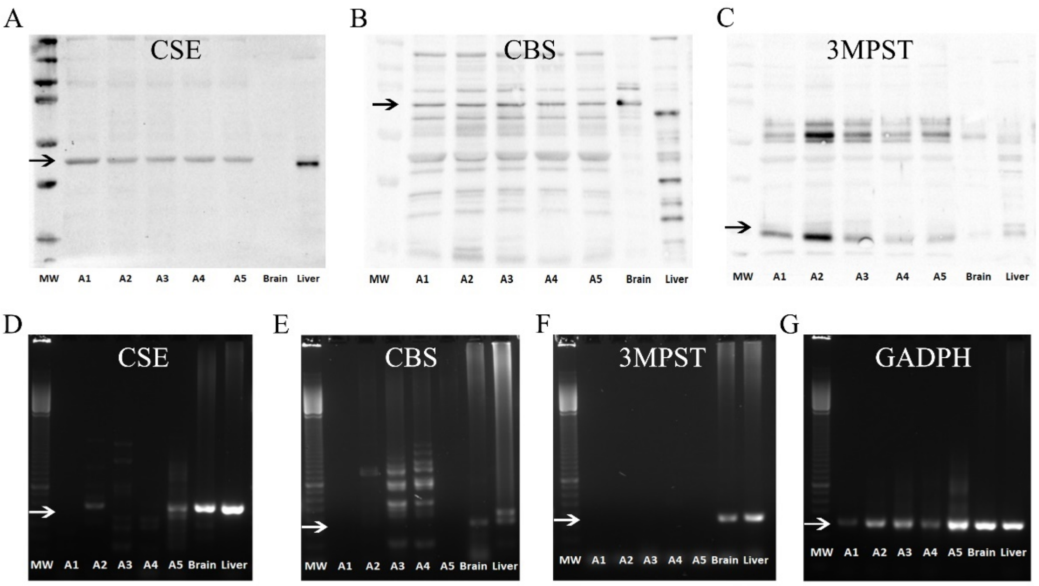

Supplementary Figure S1. Complete Western Blot membranes and RT-PCR gels. Western blot membranes pictures showing the expression of CSE (panel A), CBS (panel B) and 3MPST (panel C) in homogenates of mouse mesenteric artery, brain and liver. Panels D, E and F show the qualitative RT-PCR gels obtained for assessment of the mRNA expression of the respective enzymes in the studied tissues. Panel G: GADPH (internal control). The arrows indicate the position of the corresponding protein / DNA transcript bands.

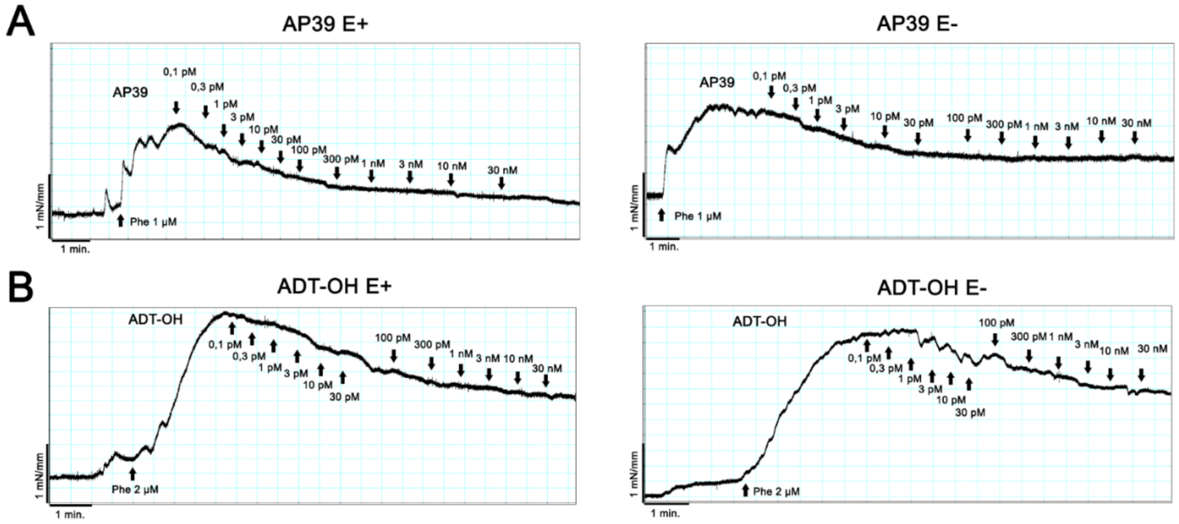

Supplementary Figure S2. Representative electronic records of the vasorelaxant responses of Phe-pre-constricted mouse mesenteric artery rings to AP39 (panel A) and ADT-OH (panel B). E+: intact vessels, E-: endothelium-denuded vessels.

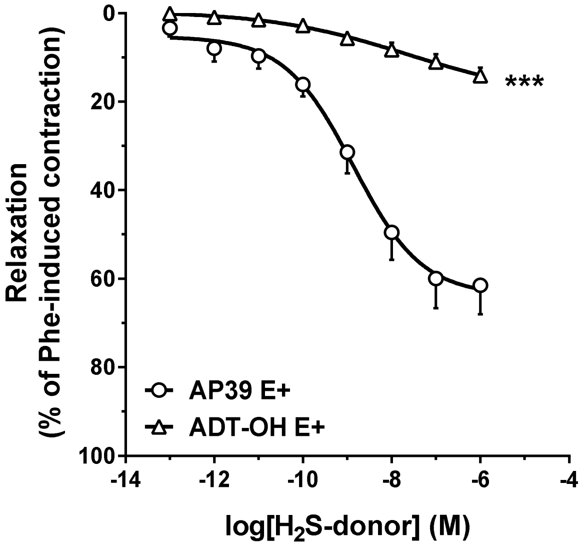

Supplementary Figure S3. Intact rat mesenteric artery ring (3rd.-order branch) response to AP39 and ADT-OH after Phe pre-contraction. ***$P<0.001$ vs. E+ E_{max} as analyzed by Student's t test.

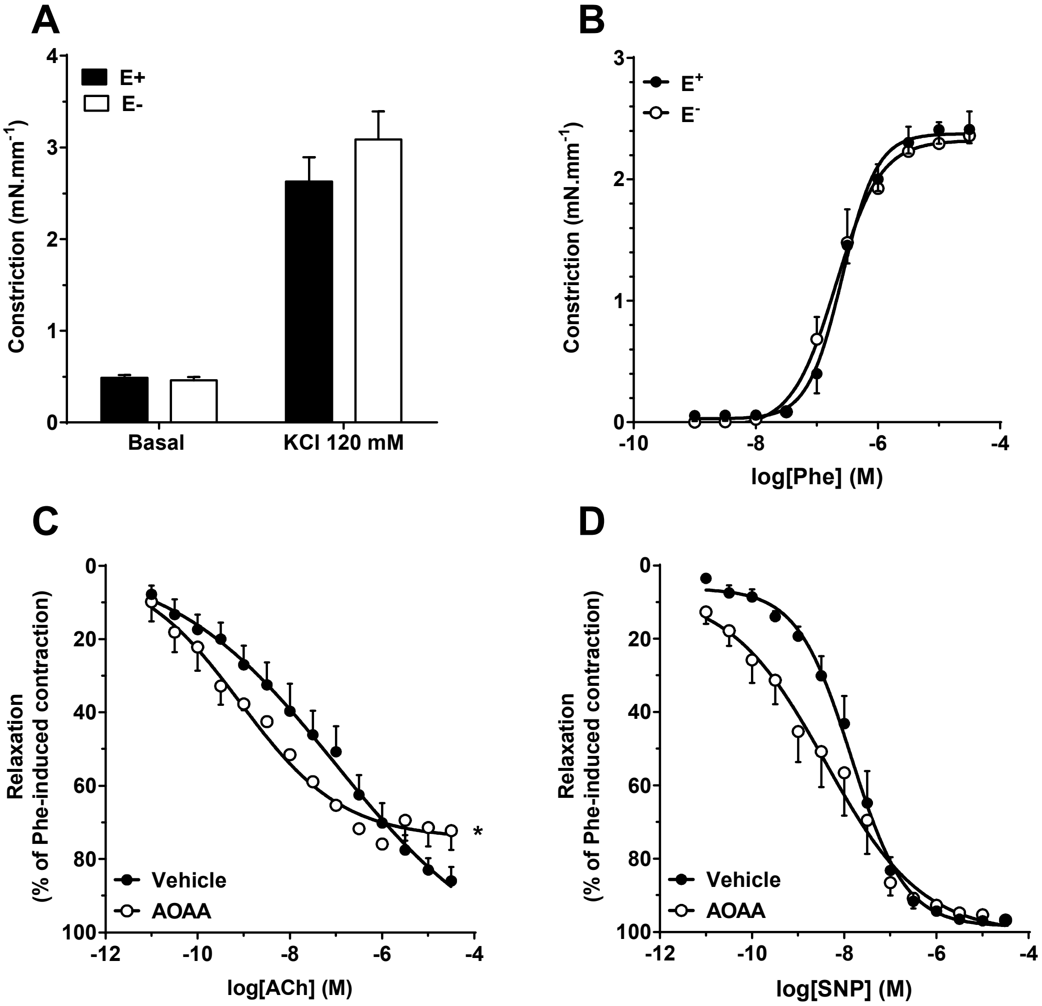

Supplementary Figure S4. Mouse mesenteric artery ring responses to different vasoactive compounds. The contractile responses of intact (E+) and endothelium-denuded vessels to 120 mM KCl (panel A) Phe (panel B) were studied. In addition, the effect of endogenous H_2S-production inhibition by 10 mM AOAA was also assessed on the vasorelaxant response of the intact vessels to acetylcholine (ACh; panel C) and sodium nitroprusside (SNP; panel D). Data expressed as mean ± S.E.M. (n=6/group). E_{max} differences expressed as *P<0.05 as analyzed by Student's t test.
